# LinearCDSfold: a tool for co-optimizing secondary structure stability and codon usage in coding sequence design

**DOI:** 10.1093/bioadv/vbag060

**Published:** 2026-02-17

**Authors:** Yu-Shen Liu, Yan-Ru Ju, Kai-Wei Chang, Chin Lung Lu

**Affiliations:** Department of Computer Science, National Tsing Hua University, Hsinchu 30013, Taiwan; Department of Computer Science, National Tsing Hua University, Hsinchu 30013, Taiwan; Department of Computer Science, National Tsing Hua University, Hsinchu 30013, Taiwan; Department of Computer Science, National Tsing Hua University, Hsinchu 30013, Taiwan

## Abstract

**Summary:**

Designing mRNA coding sequences (CDSs) for vaccine development requires co-optimizing secondary structure stability and codon usage, which are typically measured by minimum free energy (MFE) and codon adaptation index (CAI), respectively. To address this challenge, we previously employed dynamic programming and beam search techniques to develop LinearCDSfold, a tool that generates a single CDS encoding a given protein sequence by jointly optimizing MFE and CAI. It produces an exact solution with cubic-time complexity and a high-quality approximation in linear time, both with respect to the CDS length. Since reducing MFE and increasing CAI often conflict during CDS design, it is desirable to automatically generate Pareto-optimal CDSs, for which no alternative simultaneously improves both objectives. To our knowledge, DERNA is the only existing tool with this functionality. In this work, we enhance the capabilities of LinearCDSfold to automatically and efficiently generate a set of Pareto-optimal CDSs. Experiments conducted on nine protein sequences show that LinearCDSfold performs comparably to DERNA in generating Pareto-optimal CDSs while achieving substantially faster runtime.

**Availability and Implementation:**

The program of LinearCDSfold can be downloaded from https://github.com/ablab-nthu/LinearCDSfold.

## 1 Introduction

The rapid development and clinical success of messenger RNA (mRNA) vaccines against coronavirus disease 2019 (COVID-19) have convincingly demonstrated the capacity of mRNA-based therapeutics to prevent infectious diseases (Hogan and Pardi 2022). However, the intrinsic instability of mRNAs and their limited expression efficiency pose significant challenges to the delivery and protective efficacy of mRNA vaccines. A promising strategy for improving both properties involves co-optimizing secondary structure stability and codon usage in the mRNA coding sequence (CDS), which are typically quantified by minimum free energy (MFE) and codon adaptation index (CAI), respectively. In this work, we refer to this two-objective optimization task as the *CDS design problem*. Recently, several algorithms with the same computational time $O(L^{3})$ and memory usage $O(L^{2})$ have been proposed to solve the CDS design problem (Zhang *et al*. 2023, Gu *et al*. 2024, Ju *et al*. 2025), where *L* denotes the length of the CDS to be designed. Zhang *et al*. (2023) proposed the first algorithm, called LinearDesign, inspired by lattice parsing techniques from computational linguistics. To further reduce computational time, they incorporated beam search (Huang *et al*. 2019), a widely used pruning strategy, into the algorithm, enabling LinearDesign to generate high-quality approximate CDSs in linear time. Gu *et al*. (2024) later introduced the second algorithm, named DERNA, which employs a dynamic programming approach. Specifically, DERNA processes entire codons as indivisible units at each step of the algorithm, rather than handling their constituent nucleotides independently. The advantage of DERNA is that it eliminates the need to handle nucleotide dependencies across different positions within arginine and leucine codons. However, this codon-level strategy incurs a substantially higher computational cost, despite sharing the same time and space complexities as LinearDesign. In addition, DERNA enables users to identify a set of *Pareto optimal solutions* for which no other solution yields better MFE and CAI simultaneously. Recently, we proposed the third algorithm, called LinearCDSfold (Ju *et al*. 2025), which was also designed using a dynamic programming technique. In contrast to DERNA’s codon-level approach, LinearCDSfold operates on individual nucleotides at each step of its dynamic programming algorithm. The key innovation behind LinearCDSfold is a simple modification to the extended nucleotide representation used in CDSfold (Terai *et al*. 2016), enabling it to account for nucleotide dependencies within codons encoding arginine and leucine, while also integrating CAI into the new representation without introducing the inconsistencies observed by Zhang *et al*. (2023) in their study on CDSfold. One notable advantage of our LinearCDSfold is its substantially reduced computational time compared to DERNA. In addition, LinearCDSfold employed beam search in its dynamic programming algorithm, allowing for the rapid generation of high-quality approximate CDSs in linear time.

## 2 Enhanced implementation of LinearCDSfold

In fact, there exists a trade-off between decreasing the MFE and increasing the CAI when designing a CDS to encode a protein sequence. To balance the relative contributions of MFE and CAI in CDS design, LinearDesign (Zhang *et al*. 2023) defines the objective function to be minimized as $\text{MFE}-\lambda_{\text{LD}}\cdot l\cdot \;\text{log}(\text{CAI})$, where *l* denotes the number of codons in the designed CDS and $\lambda_{\text{LD}}$ is a tunable scaling parameter ranging from 0 to ∞. Note that CAI is defined as the geometric mean of the relative adaptiveness values of all codons in a CDS (Sharp and Li 1987). Hence, log(CAI) in the above objective function becomes $\frac{1}{l}$ times the sum of the logarithms of the relative adaptiveness values of individual codons. Consequently, l· log(CAI) simply equals the sum of the logarithms of the relative adaptiveness values across all codons. However, to enable efficient generation of Pareto optimal solutions using the weighted sum method, DERNA (Gu *et al*. 2024) formulates an alternative objective as $\lambda_{\text{DN}}\cdot \text{MFE}-(1-\lambda_{\text{DN}})\cdot l\cdot \;\text{log}(\text{CAI})$, with the scaling parameter $\lambda_{\text{DN}}$ ranging from 0 to 1. Previously, our implementation of LinearCDSfold (Ju *et al*. 2025) adopted the objective function from LinearDesign. In this work, we additionally incorporate the objective function proposed by DERNA, allowing LinearCDSfold to efficiently identify a set of Pareto optimal solutions using the weighted sum method (refer to the Material, available as supplementary data at *Bioinformatics Advances* online for its details).

Table 1 summarizes the key features of the three CDS design tools examined in this study—LinearDesign, DERNA, and LinearCDSfold—all of which support the co-optimization of MFE and CAI. Note that, to the best of our knowledge, the beam search functionality in the current standalone version of LinearDesign is not yet accessible to users. Moreover, LinearDesign lacks an automated mechanism for generating Pareto optimal solutions, requiring users to run the tool multiple times with manually selected $\lambda_{\text{LD}}$ values. Among these three CDS design tools, our previous study (Ju *et al*. 2025) showed that LinearDesign, DERNA, and LinearCDSfold achieved comparable accuracy in terms of both MFE and CAI when executed with exact search. In terms of computational speed, however, LinearDesign was the fastest, followed by LinearCDSfold, while DERNA was the slowest. When run with beam search, LinearCDSfold generated an approximate CDS in linear time, with high quality in terms of both MFE and CAI.

**Table 1:** Feature comparison of three CDS design tools: LinearDesign, DERNA, and LinearCDSfold.^a^

CDS design tool	MFE	CAI	Exact search	Beam search	Pareto-optimal search
LinearDesign	√	√	√	√	×
DERNA	√	√	√	×	√
LinearCDSfold	√	√	√	√	√

a For each CDS design tool, the “Exact search,” “Beam search,” and “Pareto-optimal search” columns indicate whether it can return an exactly optimal solution, a high-quality approximate solution, and a set of Pareto-optimal solutions, respectively.

## 3 Demonstration of Pareto-optimal CDS designs

LinearCDSfold was developed in C++, with its source code and usage instructions publicly accessible at https://github.com/ablab-nthu/LinearCDSfold. Below, we assess the capability of LinearCDSfold to generate Pareto-optimal CDSs that achieve different trade-offs between CAI and MFE, using nine protein sequences selected from the UniProt dataset (Bateman *et al*. 2023) and the codon usage frequency of Homo sapiens from the Codon Usage Database (Nakamura *et al*. 2000). We then compare its results with those obtained by DERNA. This experiment was conducted on a Linux-based PC equipped with a 4.4 GHz CPU and 128 GB of RAM, with both LinearCDSfold and DERNA executed using their default parameter settings.

**Figure 1:**
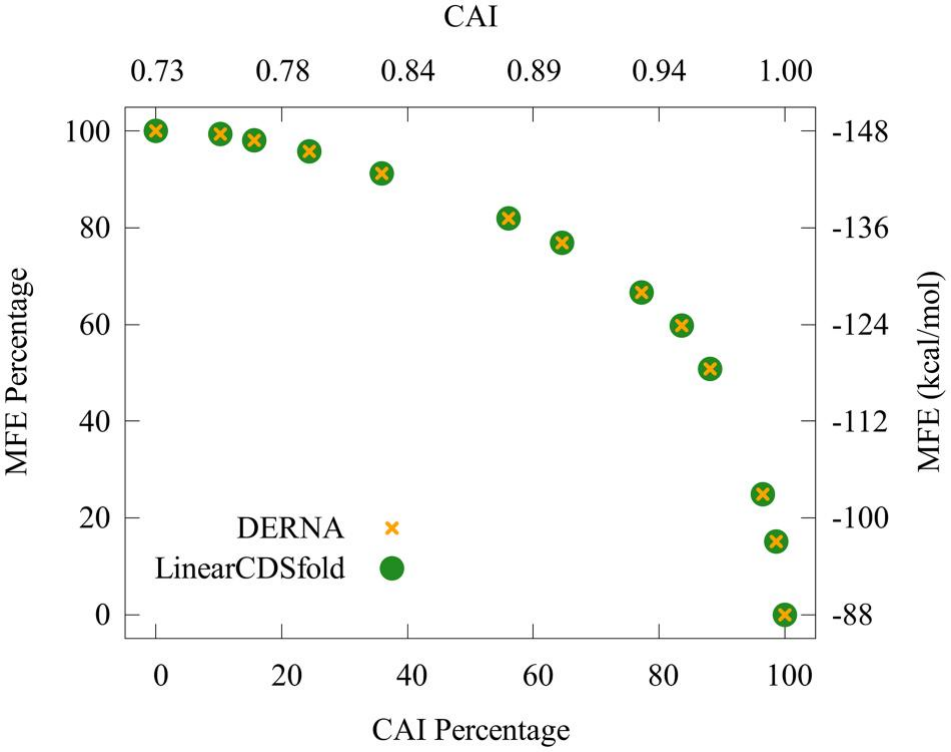
Comparison of Pareto-optimal CDSs generated by DERNA and LinearCDSfold for UniProt sequence P15421. The right *y*-axis indicates MFE, and the left *y*-axis represents its range-normalized MFE percentage. Similarly, the top *x*-axis displays CAI, and the bottom *x*-axis shows its range-normalized CAI percentage.

As shown in Table 2, for each of the nine protein sequences tested, LinearCDSfold and DERNA generated not only the same total number of Pareto-optimal CDSs but also identical counts of distinct solutions. These results suggest that the two tools exhibit comparable design capability under the examined dataset. To further illustrate this, a representative protein sequence (UniProt ID: P15421) was selected, and the Pareto fronts generated by LinearCDSfold and DERNA were plotted with CAI on the *x*-axis and MFE on the *y*-axis. To enable comparison across Pareto-optimal CDSs, raw MFE values were linearly scaled to a normalized range from 0% to 100%, based on the minimum and maximum MFE values observed within the set. An MFE percentage of 100% corresponds to the most stable (i.e. best) MFE among all Pareto-optimal CDSs, while 0% corresponds to the least stable (i.e. worst) MFE. Following the methodology used in DERNA (Gu *et al*. 2024), these normalized MFE values are referred to as *MFE percentages*. Similarly, *CAI percentages* were defined analogously, based on the maximum and minimum CAI values observed within the Pareto-optimal CDS set. As illustrated in Fig. 1, both LinearCDSfold and DERNA produced the same set of 13 distinct Pareto-optimal CDSs. In contrast, the results in Table 2 further indicate that the only difference observed between DERNA and LinearCDSfold was in computational efficiency. LinearCDSfold consistently outperformed DERNA, achieving speedups ranging from 4.9-fold to 8.6-fold. This performance difference was associated with protein sequence length and amino acid composition. For detailed results of the experiment conducted in this study, please refer to the Material, available as supplementary data at *Bioinformatics Advances* online.

**Table 2:** Comparison of Pareto-optimal CDSs generated by DERNA and LinearCDSfold.

		Distinct Pareto-optimal CDSs (n/N)^a^			Total runtime (min)		
UniProt ID	Length (aa)	DERNA	LinearCDSfold	Common CDSs^b^	DERNA	LinearCDSfold	Runtime ratio^c^
P15421	78	13/25	13/25	13	10.4	1.2	8.6
Q6IUF9	94	12/24	12/24	12	7.6	1.2	6.1
B0BLK7	99	11/24	11/24	11	12.4	1.9	6.7
Q27YE2	101	13/29	13/29	13	14.5	2.3	6.4
P9WN84	130	11/20	11/20	11	22.8	3.3	7.0
P0AC51	171	18/44	18/44	18	56.2	9.3	6.1
Q8VIL3	266	13/22	13/22	13	59.6	11.6	5.1
O95229	277	14/28	14/28	14	85.6	16.1	5.3
Q2TBH8	286	19/37	19/37	19	103.0	20.9	4.9

a n/N denotes the number of distinct Pareto-optimal CDSs (*n*) out of the total Pareto-optimal CDSs (*N*) generated by each tool.

b “Common CDSs” indicates the number of distinct Pareto-optimal CDSs shared by both DERNA and LinearCDSfold.

c Runtime ratio represents the relative runtime of DERNA compared to LinearCDSfold.

## Supplementary Material

vbag060_Supplementary_Data

## Data Availability

All protein sequence data used in this study are available at https://github.com/ablab-nthu/LinearCDSfold.
