# Supplementary material for: LinearCDSfold: a tool for co-optimizing secondary structure stability and codon usage in coding sequence design
Source: Bioinform Adv. 2026 Feb 17;6(1):vbag060. doi: 10.1093/bioadv/vbag060 (PMC12955848; doi:10.1093/bioadv/vbag060)
Supplement: vbag060_Supplementary_Data [file vbag060_supplementary_data.pdf]

Supplementary Material for

LinearCDSfold: a tool for co-optimizing secondary structure stability and codon usage in coding sequence design

Yu-Shen Liu[1] Yan-Ru Ju[1] Kai-Wei Chang[1] Chin Lung Lu[1,*]

[1]Department of Computer Science, National Tsing Hua University, Hsinchu 30013, Taiwan

1 Algorithm for identifying Pareto-optimal CDSs

Following the DERNA study (1), we apply the weighted sum method to identify a set of Pareto-optimal coding sequences (CDSs) for a given protein by optimizing distinct convex combinations of minimum free energy (MFE) and codon adaptation index (CAI). This approach to enumerating Pareto-optimal CDSs is equivalent to solving multiple instances of the CDS design problem, each corresponding to a different value of the scaling parameter $\lambda_{\text{DN}} \in [0, 1]$. Subsequently, for each instance, we further use our dynamic programming algorithm (2) to compute an optimal CDS. For this purpose, we maintain a queue Q containing intervals of the form $[\lambda_l, \lambda_r]$, where $0 \leq \lambda_l < \lambda_r \leq 1$, and a hash table H in which each entry $H[\lambda_{\text{DN}}]$ corresponds to the optimal solution for the CDS design problem under the scaling parameter λ_{DN}.

The algorithm we use to generate Pareto-optimal CDSs in our LinearCDSfold is described as follows. We initialize Q with the interval $[\epsilon, 1 - \epsilon]$, where ϵ is a small positive constant with a default value of 10^{-5}. In addition, we initialize $H[\epsilon]$ and $H[1 - \epsilon]$ with the optimal solutions for the CDS design problem at scaling parameters ϵ and $1 - \epsilon$, respectively. As long as Q is not empty, we first extract an interval $[\lambda_l, \lambda_r]$ from Q, then use our dynamic programming algorithm (2) to solve the CDS design problem at the midpoint scaling parameter $\lambda_m = (\lambda_l + \lambda_r)/2$, and finally store the resulting optimal solution in $H[\lambda_m]$. Next, we consider the following two cases.

*To whom correspondence should be addressed. Email: cllu@cs.nthu.edu.tw

Case 1: Suppose that $\lambda_m \geq \tau_1$, where τ_1 is a tunable threshold parameter with a default value of 0.0025. If $H[\lambda_m] \neq H[\lambda_l]$ and $\lambda_m - \lambda_l \geq \tau_1$, then we add the interval $[\lambda_l, \lambda_m]$ to Q. In addition, if $H[\lambda_m] \neq H[\lambda_r]$ and $\lambda_r - \lambda_m \geq \tau_1$, then we add the interval $[\lambda_m, \lambda_r]$ to Q. **Case 2:** Suppose that $\lambda_m < \tau_1$. Let τ_2 be another tunable threshold parameter, with a default value of 0.00075. If $H[\lambda_m] \neq H[\lambda_l]$ and $\lambda_m - \lambda_l \geq \tau_2$, then we add the interval $[\lambda_l, \lambda_m]$ to Q. Similarly, if $H[\lambda_m] \neq H[\lambda_r]$ and $\lambda_r - \lambda_m \geq \tau_2$, then we add the interval $[\lambda_m, \lambda_r]$ to Q.

The weighted sum method described above is functionally equivalent to the approach used in DERNA. Although the DERNA manuscript primarily describes a single-threshold method with the tunable threshold parameter τ, its software implementation utilizes two distinct parameters, τ and τ_2 (controllable via the `-t` and `-p` options, respectively), to better address the non-uniform sampling issue inherent in the weighted sum method. While a single-parameter approach can recover missing Pareto-optimal solutions by reducing τ, it results in a disproportionate increase in the number of λ values to explore, many of which lead to redundant solutions. To maintain consistency with the DERNA implementation and to ensure a more complete Pareto front without excessive computational overhead, LinearCDSFold adopts this dual-parameter approach using τ_1 and τ_2 (controllable via the `-t` and `-u` options, respectively).

2 Experimental results

2.1 Effect of reducing threshold parameter τ_1

As observed in the DERNA study (1), the weighted sum method can yield the same Pareto optimal solutions even when different values of λ_{DN} are used. However, by reducing the default value of τ_1 or τ_2, our LinearCDSfold can explore a greater number of distinct λ_{DN} values, potentially yielding more Pareto-optimal CDSs. Using the protein sequence (UniProt ID: P15421) as an example, if the default value of τ_1 is lowered to 0.0001, both LinearCDSfold and DERNA explore 101 distinct λ_{DN} values and, as a result, identify the same set of 21 distinct Pareto-optimal CDSs, with total running times of 293 seconds and 2,530 seconds, respectively, as shown in Figure S1. This outcome includes 8 additional Pareto-optimal CDSs compared to the result obtained using the default value of τ_1, which yielded 13 distinct Pareto-optimal CDSs by examining 25 different λ_{DN} values as shown in Figure 1 of the main text. However, for this outcome, our LinearCDSfold is still significantly faster than DERNA.

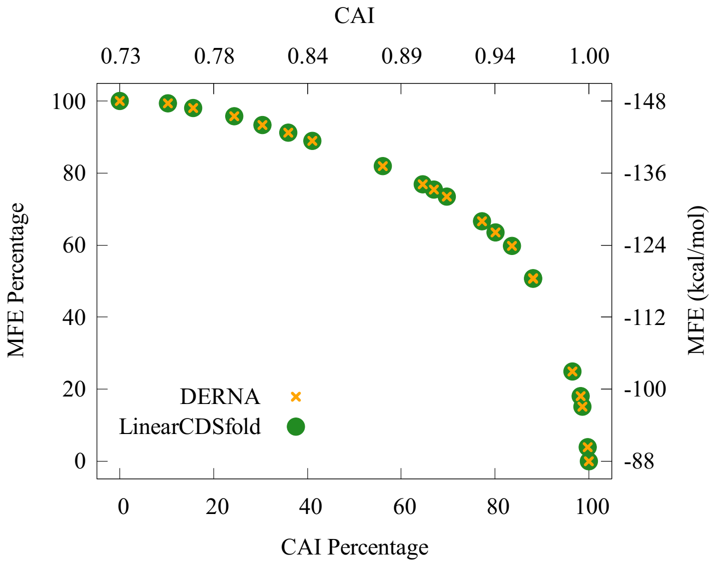

Figure S1: Comparison of Pareto-optimal CDSs produced by DERNA and LinearCDSfold for UniProt sequence P15421 under $\tau_1 = 0.0001$. The right y-axis indicates MFE and the left y-axis represents its range-normalized MFE percentage. Similarly, the top x-axis displays CAI and the bottom x-axis shows its range-normalized CAI percentage.

2.2 Tabulated results for all tested protein sequences

The tables in the following summarize the experimental results of Pareto-optimal CDSs generated using two CDS design tools, DERNA and LinearCDSfold, applied to nine protein sequences analyzed in this study. For each Pareto-optimal CDS, the values of minimum free energy (MFE), codon adaptation index (CAI), and computational runtime are reported. Because these tables include repeated Pareto-optimal CDSs, each is immediately followed by a companion table presenting the corresponding set of distinct Pareto-optimal CDSs.

Table S1: Pareto-optimal CDSs generated by DERNA and LinearCDSfold for protein sequence P15421 (78 amino acids). Both methods explored 25 λ_{DN} values and yielded the same set of 13 distinct Pareto-optimal CDSs (see Supplementary Table S2). The total exploration times were 625 seconds for DERNA and 73 seconds for LinearCDSfold.

	DERNA				LinearCDSfold			
Index[a]	λ_{DN}	MFE	CAI	Runtime (sec)	λ_{DN}	MFE	CAI	Runtime (sec)
1	0.000010	-88.0	1.000	25.0	0.000010	-88.0	1.000	2.9
2	0.999990	-148.7	0.734	25.0	0.999990	-148.7	0.734	2.9
3	0.500000	-148.7	0.734	25.0	0.500000	-148.7	0.734	2.9
4	0.250005	-148.7	0.734	25.0	0.250005	-148.7	0.734	2.9
5	0.125007	-148.7	0.734	25.0	0.125007	-148.7	0.734	2.9
6	0.062509	-148.3	0.761	25.0	0.062509	-148.3	0.761	2.9
7	0.031259	-148.3	0.761	25.0	0.031259	-148.3	0.761	2.9
8	0.093758	-148.7	0.734	25.0	0.093758	-148.7	0.734	2.9
9	0.015635	-146.1	0.799	25.0	0.015635	-146.1	0.799	2.9
10	0.078133	-148.7	0.734	25.0	0.078133	-148.7	0.734	2.9
11	0.007822	-137.7	0.883	25.0	0.007822	-137.7	0.883	2.9
12	0.023447	-148.3	0.761	25.0	0.023447	-148.3	0.761	2.9
13	0.070321	-148.7	0.734	25.0	0.070321	-148.7	0.734	2.9
14	0.003916	-128.4	0.940	25.0	0.003916	-128.4	0.940	2.9
15	0.011729	-146.1	0.799	25.0	0.011729	-146.1	0.799	2.9
16	0.019541	-148.3	0.761	25.0	0.019541	-148.3	0.761	2.9
17	0.066415	-148.7	0.734	25.0	0.066415	-148.7	0.734	2.9
18	0.001963	-124.3	0.956	25.0	0.001963	-124.3	0.956	2.9
19	0.005869	-134.7	0.906	25.0	0.005869	-134.7	0.906	2.9
20	0.009775	-143.4	0.829	25.0	0.009775	-143.4	0.829	2.9
21	0.017588	-147.5	0.775	25.0	0.017588	-147.5	0.775	2.9
22	0.064462	-148.3	0.761	25.0	0.064462	-148.3	0.761	2.9
23	0.000987	-103.1	0.991	25.0	0.000987	-103.1	0.991	2.9
24	0.000498	-97.2	0.997	25.0	0.000498	-97.2	0.997	2.9
25	0.001475	-118.8	0.968	25.0	0.001475	-118.8	0.968	2.9

[a] Index denotes the sequential numbering of λ_{DN} values generated by the tool.

Table S2: Distinct Pareto-optimal CDSs generated by DERNA and LinearCDSfold for protein sequence P15421. Both methods yielded the same set of 13 distinct Pareto-optimal CDSs.

	DERNA				LinearCDSfold			
Number	MFE	CAI	MFE (%)[a]	CAI (%)[b]	MFE	CAI	MFE (%)	CAI (%)
1	-88.0	1.000	0	100	-88.0	1.000	0	100
2	-148.7	0.734	100	0	-148.7	0.734	100	0
3	-148.3	0.761	99	10	-148.3	0.761	99	10
4	-146.1	0.799	96	24	-146.1	0.799	96	24
5	-137.7	0.883	82	56	-137.7	0.883	82	56
6	-128.4	0.940	67	77	-128.4	0.940	67	77
7	-124.3	0.956	60	84	-124.3	0.956	60	84
8	-134.7	0.906	77	65	-134.7	0.906	77	65
9	-143.4	0.829	91	36	-143.4	0.829	91	36
10	-147.5	0.775	98	16	-147.5	0.775	98	16
11	-103.1	0.991	25	97	-103.1	0.991	25	97
12	-97.2	0.997	15	99	-97.2	0.997	15	99
13	-118.8	0.968	51	88	-118.8	0.968	51	88

[a] MFE (%) inditicates the MFE percentage.
[b] CAI (%) inditicates the CAI percentage.

Table S3: Pareto-optimal CDSs generated by DERNA and LinearCDSfold for protein sequence Q6IUF9 (94 amino acids). Both methods explored 24 λ_{DN} values and yielded the same set of 12 distinct Pareto-optimal CDSs (see Supplementary Table S4). The total exploration times were 456 seconds for DERNA and 74 seconds for LinearCDSfold.

	DERNA				LinearCDSfold			
Index	λ_{DN}	MFE	CAI	Runtime (sec)	λ_{DN}	MFE	CAI	Runtime (sec)
1	0.000010	-76.2	1.000	19.0	0.000010	-76.2	1.000	3.1
2	0.999990	-171.8	0.697	19.0	0.999990	-171.8	0.697	3.1
3	0.500000	-171.8	0.697	19.0	0.500000	-171.8	0.697	3.1
4	0.250005	-171.8	0.697	19.0	0.250005	-171.8	0.697	3.1
5	0.125007	-171.8	0.697	19.0	0.125007	-171.8	0.697	3.1
6	0.062509	-171.8	0.697	19.0	0.062509	-171.8	0.697	3.1
7	0.031259	-169.6	0.770	19.0	0.031259	-169.6	0.770	3.1
8	0.015635	-168.9	0.779	19.0	0.015635	-168.9	0.779	3.1
9	0.046884	-171.8	0.697	19.0	0.046884	-171.8	0.697	3.1
10	0.007822	-163.8	0.817	19.0	0.007822	-163.8	0.817	3.1
11	0.023447	-169.6	0.770	19.0	0.023447	-169.6	0.770	3.1
12	0.039072	-169.6	0.770	19.0	0.039072	-169.6	0.770	3.1
13	0.003916	-146.4	0.906	19.0	0.003916	-146.4	0.906	3.1
14	0.011729	-168.9	0.779	19.0	0.011729	-168.9	0.779	3.1
15	0.019541	-169.6	0.770	19.0	0.019541	-169.6	0.770	3.1
16	0.042978	-171.8	0.697	19.0	0.042978	-171.8	0.697	3.1
17	0.001963	-132.0	0.938	19.0	0.001963	-132.0	0.938	3.1
18	0.005869	-154.3	0.870	19.0	0.005869	-154.3	0.870	3.1
19	0.009775	-168.5	0.783	19.0	0.009775	-168.5	0.783	3.1
20	0.017588	-169.6	0.770	19.0	0.017588	-169.6	0.770	3.1
21	0.041025	-171.8	0.697	19.0	0.041025	-171.8	0.697	3.1
22	0.000987	-91.3	0.994	19.0	0.000987	-91.3	0.994	3.1
23	0.000498	-86.2	0.997	19.0	0.000498	-86.2	0.997	3.1
24	0.001475	-117.4	0.962	19.0	0.001475	-117.4	0.962	3.1

Table S4: Distinct Pareto-optimal CDSs generated by DERNA and LinearCDSfold for protein sequence Q6IUF9. Both methods yielded the same set of 12 distinct Pareto-optimal CDSs.

	DERNA				LinearCDSfold			
Number	MFE	CAI	MFE (%)	CAI (%)	MFE	CAI	MFE (%)	CAI (%)
1	-76.2	1.000	0	100	-76.2	1.000	0	100
2	-171.8	0.697	100	0	-171.8	0.697	100	0
3	-169.6	0.770	98	24	-169.6	0.770	98	24
4	-168.9	0.779	97	27	-168.9	0.779	97	27
5	-163.8	0.817	92	40	-163.8	0.817	92	40
6	-146.4	0.906	73	69	-146.4	0.906	73	69
7	-132.0	0.938	58	79	-132.0	0.938	58	79
8	-154.3	0.870	82	57	-154.3	0.870	82	57
9	-168.5	0.783	97	28	-168.5	0.783	97	28
10	-91.3	0.994	16	98	-91.3	0.994	16	98
11	-86.2	0.997	10	99	-86.2	0.997	10	99
12	-117.4	0.962	43	87	-117.4	0.962	43	87

Table S5: Pareto-optimal CDSs generated by DERNA and LinearCDSfold for protein sequence B0BLK7 (99 amino acids). Both methods explored 24 λ_{DN} values and yielded the same set of 11 distinct Pareto-optimal CDSs (see Supplementary Table S6). The total exploration times were 744 seconds for DERNA and 111 seconds for LinearCDSfold.

	DERNA				LinearCDSfold			
Index	λ_{DN}	MFE	CAI	Runtime (sec)	λ_{DN}	MFE	CAI	Runtime (sec)
1	0.000010	-100.0	1.000	31.0	0.000010	-100.0	1.000	4.6
2	0.999990	-187.7	0.764	31.0	0.999990	-187.7	0.764	4.6
3	0.500000	-187.7	0.764	31.0	0.500000	-187.7	0.764	4.6
4	0.250005	-187.7	0.764	31.0	0.250005	-187.7	0.764	4.6
5	0.125007	-187.7	0.764	31.0	0.125007	-187.7	0.764	4.6
6	0.062509	-187.7	0.764	31.0	0.062509	-187.7	0.764	4.6
7	0.031259	-187.3	0.783	31.0	0.031259	-187.3	0.783	4.6
8	0.015635	-186.4	0.796	31.0	0.015635	-186.4	0.796	4.6
9	0.046884	-187.3	0.783	31.0	0.046884	-187.3	0.783	4.7
10	0.007822	-177.7	0.884	31.0	0.007822	-177.7	0.884	4.6
11	0.023447	-187.3	0.783	31.0	0.023447	-187.3	0.783	4.6
12	0.054696	-187.3	0.783	31.0	0.054696	-187.3	0.783	4.6
13	0.003916	-177.7	0.884	31.0	0.003916	-177.7	0.884	4.6
14	0.011729	-181.9	0.849	31.0	0.011729	-181.9	0.849	4.7
15	0.019541	-187.3	0.783	31.0	0.019541	-187.3	0.783	4.7
16	0.058603	-187.7	0.764	31.0	0.058603	-187.7	0.764	4.7
17	0.001963	-154.9	0.953	31.0	0.001963	-154.9	0.953	4.6
18	0.009775	-181.9	0.849	31.0	0.009775	-181.9	0.849	4.6
19	0.013682	-183.2	0.835	31.0	0.013682	-183.2	0.835	4.6
20	0.017588	-186.4	0.796	31.0	0.017588	-186.4	0.796	4.7
21	0.056650	-187.3	0.783	31.0	0.056649	-187.3	0.783	4.7
22	0.000987	-129.3	0.987	31.0	0.000987	-129.3	0.987	4.6
23	0.000498	-119.3	0.995	31.0	0.000498	-119.3	0.995	4.6
24	0.001475	-142.9	0.971	31.0	0.001475	-142.9	0.971	4.6

Table S6: Distinct Pareto-optimal CDSs generated by DERNA and LinearCDSfold for protein sequence B0BLK7. Both methods yielded the same set of 11 distinct Pareto-optimal CDSs.

	DERNA				LinearCDSfold			
Number	MFE	CAI	MFE (%)	CAI (%)	MFE	CAI	MFE (%)	CAI (%)
1	-100.0	1.000	0	100	-100.0	1.000	0	100
2	-187.7	0.764	100	0	-187.7	0.764	100	0
3	-187.3	0.783	100	8	-187.3	0.783	100	8
4	-186.4	0.796	99	14	-186.4	0.796	99	14
5	-177.7	0.884	89	51	-177.7	0.884	89	51
6	-181.9	0.849	93	36	-181.9	0.849	93	36
7	-154.9	0.953	63	80	-154.9	0.953	63	80
8	-183.2	0.835	95	30	-183.2	0.835	95	30
9	-129.3	0.987	33	94	-129.3	0.987	33	94
10	-119.3	0.995	22	98	-119.3	0.995	22	98
11	-142.9	0.971	49	88	-142.9	0.971	49	88

Table S7: Pareto-optimal CDSs generated by DERNA and LinearCDSfold for protein sequence Q27YE2 (101 amino acids). Both methods explored 29 λ_{DN} values and yielded the same set of 13 distinct Pareto-optimal CDSs (see Supplementary Table S8). The total exploration times were 870 seconds for DERNA and 136 seconds for LinearCDSfold.

	DERNA				LinearCDSfold			
Index	λ_{DN}	MFE	CAI	Runtime (sec)	λ_{DN}	MFE	CAI	Runtime (sec)
1	0.000010	-88.9	1.000	30.0	0.000010	-88.9	1.000	4.7
2	0.999990	-183.8	0.777	30.0	0.999990	-183.8	0.777	4.7
3	0.500000	-183.8	0.777	30.0	0.500000	-183.8	0.777	4.7
4	0.250005	-183.8	0.777	30.0	0.250005	-183.8	0.777	4.7
5	0.125007	-183.8	0.777	30.0	0.125007	-183.8	0.777	4.7
6	0.062509	-183.7	0.785	30.0	0.062509	-183.7	0.785	4.7
7	0.031259	-183.5	0.796	30.0	0.031259	-183.5	0.796	4.7
8	0.093758	-183.7	0.785	30.0	0.093758	-183.7	0.785	4.7
9	0.015635	-182.2	0.814	30.0	0.015635	-182.2	0.814	4.7
10	0.046884	-183.5	0.796	30.0	0.046884	-183.5	0.796	4.7
11	0.109383	-183.8	0.777	30.0	0.109383	-183.8	0.777	4.7
12	0.007822	-178.9	0.839	30.0	0.007822	-178.9	0.839	4.7
13	0.023447	-183.5	0.796	30.0	0.023447	-183.5	0.796	4.7
14	0.054696	-183.5	0.796	30.0	0.054696	-183.5	0.796	4.7
15	0.101570	-183.8	0.777	30.0	0.101570	-183.8	0.777	4.7
16	0.003916	-161.4	0.915	30.0	0.003916	-161.4	0.915	4.7
17	0.011729	-182.2	0.814	30.0	0.011729	-182.2	0.814	4.7
18	0.019541	-183.5	0.796	30.0	0.019541	-183.5	0.796	4.7
19	0.058603	-183.5	0.796	30.0	0.058603	-183.5	0.796	4.7
20	0.097664	-183.7	0.785	30.0	0.097664	-183.7	0.785	4.7
21	0.001963	-144.2	0.959	30.0	0.001963	-144.2	0.959	4.7
22	0.005869	-175.4	0.858	30.0	0.005869	-175.4	0.858	4.7
23	0.009775	-180.9	0.826	30.0	0.009775	-180.9	0.826	4.7
24	0.017588	-183.5	0.796	30.0	0.017588	-183.5	0.796	4.7
25	0.060556	-183.5	0.796	30.0	0.060556	-183.5	0.796	4.7
26	0.099617	-183.8	0.777	30.0	0.099617	-183.8	0.777	4.7
27	0.000987	-126.5	0.979	30.0	0.000987	-126.5	0.979	4.7
28	0.000498	-98.7	0.997	30.0	0.000498	-98.7	0.997	4.7
29	0.001475	-141.5	0.963	30.0	0.001475	-141.5	0.963	4.7

Table S8: Distinct Pareto-optimal CDSs generated by DERNA and LinearCDSfold for protein sequence Q27YE2. Both methods yielded the same set of 13 distinct Pareto-optimal CDSs.

	DERNA				LinearCDSfold			
Number	MFE	CAI	MFE (%)	CAI (%)	MFE	CAI	MFE (%)	CAI (%)
1	-88.9	1.000	0	100	-88.9	1.000	0	100
2	-183.8	0.777	100	0	-183.8	0.777	100	0
3	-183.7	0.785	100	4	-183.7	0.785	100	4
4	-183.5	0.796	100	8	-183.5	0.796	100	8
5	-182.2	0.814	98	17	-182.2	0.814	98	17
6	-178.9	0.839	95	28	-178.9	0.839	95	28
7	-161.4	0.915	76	62	-161.4	0.915	76	62
8	-144.2	0.959	58	81	-144.2	0.959	58	81
9	-175.4	0.858	91	36	-175.4	0.858	91	36
10	-180.9	0.826	97	22	-180.9	0.826	97	22
11	-126.5	0.979	40	91	-126.5	0.979	40	91
12	-98.7	0.997	10	99	-98.7	0.997	10	99
13	-141.5	0.963	55	83	-141.5	0.963	55	83

Table S9: Pareto-optimal CDSs generated by DERNA and LinearCDSfold for protein sequence P9WN84 (130 amino acids). Both methods explored 20 λ_{DN} values and yielded the same set of 11 distinct Pareto-optimal CDSs (see Supplementary Table S10). The total exploration times were 1,369 seconds for DERNA and 196 seconds for LinearCDSfold.

	DERNA				LinearCDSfold			
Index	λ_{DN}	MFE	CAI	Runtime (sec)	λ_{DN}	MFE	CAI	Runtime (sec)
1	0.000010	-169.7	1.000	68.0	0.000010	-169.7	1.000	9.7
2	0.999990	-285.1	0.779	69.0	0.999990	-285.1	0.779	9.8
3	0.500000	-285.1	0.779	68.0	0.500000	-285.1	0.779	9.8
4	0.250005	-285.1	0.779	69.0	0.250005	-285.1	0.779	9.8
5	0.125007	-285.1	0.779	68.0	0.125007	-285.1	0.779	9.8
6	0.062509	-285.1	0.779	69.0	0.062509	-285.1	0.779	9.8
7	0.031259	-285.1	0.779	68.0	0.031259	-285.1	0.779	9.8
8	0.015635	-282.3	0.819	69.0	0.015635	-282.3	0.819	9.8
9	0.007822	-274.2	0.871	69.0	0.007822	-274.2	0.871	9.8
10	0.023447	-285.1	0.779	68.0	0.023447	-285.1	0.779	9.8
11	0.003916	-271.8	0.882	69.0	0.003916	-271.8	0.882	9.8
12	0.011729	-282.3	0.819	69.0	0.011729	-282.3	0.819	9.8
13	0.019541	-282.3	0.819	68.0	0.019541	-282.3	0.819	9.8
14	0.001963	-237.6	0.950	68.0	0.001963	-237.6	0.950	9.8
15	0.005869	-272.5	0.880	69.0	0.005869	-272.5	0.880	9.8
16	0.009775	-280.5	0.832	69.0	0.009775	-280.5	0.832	9.8
17	0.021494	-282.3	0.819	68.0	0.021494	-282.3	0.819	9.8
18	0.000987	-205.9	0.987	68.0	0.000987	-205.9	0.987	9.8
19	0.000498	-193.4	0.994	68.0	0.000498	-193.4	0.994	9.8
20	0.001475	-214.1	0.979	68.0	0.001475	-214.1	0.979	9.7

Table S10: Distinct Pareto-optimal CDSs generated by DERNA and LinearCDSfold for protein sequence P9WN84. Both methods yielded the same set of 11 distinct Pareto-optimal CDSs.

	DERNA				LinearCDSfold			
Number	MFE	CAI	MFE (%)	CAI (%)	MFE	CAI	MFE (%)	CAI (%)
1	-169.7	1.000	0	100	-169.7	1.000	0	100
2	-285.1	0.779	100	0	-285.1	0.779	100	0
3	-282.3	0.819	98	18	-282.3	0.819	98	18
4	-274.2	0.871	91	42	-274.2	0.871	91	42
5	-271.8	0.882	88	47	-271.8	0.882	88	47
6	-237.6	0.950	59	77	-237.6	0.950	59	77
7	-272.5	0.880	89	46	-272.5	0.880	89	46
8	-280.5	0.832	96	24	-280.5	0.832	96	24
9	-205.9	0.987	31	94	-205.9	0.987	31	94
10	-193.4	0.994	21	97	-193.4	0.994	21	97
11	-214.1	0.979	38	90	-214.1	0.979	38	90

Table S11: Pareto-optimal CDSs generated by DERNA and LinearCDSfold for protein sequence P0AC51 (171 amino acids). Both methods explored 44 λ_{DN} values and yielded the same set of 18 distinct Pareto-optimal CDSs (see Supplementary Table S12). The total exploration times were 3,374 seconds for DERNA and 556 seconds for LinearCDSfold.

	DERNA				LinearCDSfold			
Index	λ_{DN}	MFE	CAI	Runtime (sec)	λ_{DN}	MFE	CAI	Runtime (sec)
1	0.000010	-225.5	1.000	76.0	0.000010	-225.5	1.000	12.5
2	0.999990	-353.4	0.726	77.0	0.999990	-353.4	0.726	12.6
3	0.500000	-353.4	0.726	77.0	0.500000	-353.4	0.726	12.6
4	0.250005	-353.4	0.726	77.0	0.250005	-353.4	0.726	12.7
5	0.125007	-353.2	0.740	77.0	0.125007	-353.2	0.740	12.7
6	0.062509	-352.9	0.751	77.0	0.062509	-352.9	0.751	12.6
7	0.187506	-353.4	0.726	77.0	0.187506	-353.4	0.726	12.7
8	0.031259	-352.2	0.767	77.0	0.031259	-352.2	0.767	12.6
9	0.093758	-353.2	0.740	77.0	0.093758	-353.2	0.740	12.6
10	0.156257	-353.4	0.726	77.0	0.156257	-353.4	0.726	12.7
11	0.015635	-350.7	0.781	76.0	0.015635	-350.7	0.781	12.7
12	0.046884	-352.6	0.760	77.0	0.046884	-352.6	0.760	12.7
13	0.078133	-353.0	0.748	77.0	0.078133	-353.0	0.748	12.6
14	0.140632	-353.4	0.726	77.0	0.140632	-353.4	0.726	12.7
15	0.007822	-340.5	0.846	76.0	0.007822	-340.5	0.846	12.6
16	0.023447	-351.7	0.773	76.0	0.023447	-351.7	0.773	12.7
17	0.039072	-352.6	0.760	77.0	0.039072	-352.6	0.760	12.7
18	0.054696	-352.6	0.760	77.0	0.054696	-352.6	0.760	12.7
19	0.070321	-352.9	0.751	77.0	0.070321	-352.9	0.751	12.7
20	0.085946	-353.2	0.740	77.0	0.085946	-353.2	0.740	12.7
21	0.132820	-353.2	0.740	77.0	0.132820	-353.2	0.740	12.7
22	0.003916	-318.5	0.908	76.0	0.003916	-318.5	0.908	12.6
23	0.011729	-342.6	0.836	77.0	0.011729	-342.6	0.836	12.6
24	0.019541	-351.7	0.773	76.0	0.019541	-351.7	0.773	12.6
25	0.027353	-351.7	0.773	76.0	0.027353	-351.7	0.773	12.7
26	0.035166	-352.2	0.767	77.0	0.035166	-352.2	0.767	12.6
27	0.058603	-352.6	0.760	77.0	0.058603	-352.6	0.760	12.6
28	0.074227	-353.0	0.748	77.0	0.074227	-353.0	0.748	12.6
29	0.082040	-353.2	0.740	77.0	0.082040	-353.2	0.740	12.6
30	0.136726	-353.2	0.740	77.0	0.136726	-353.2	0.740	12.7
31	0.001963	-285.8	0.963	76.0	0.001963	-285.8	0.963	12.6
32	0.005869	-336.1	0.860	76.0	0.005869	-336.1	0.860	12.6
33	0.009775	-341.7	0.841	76.0	0.009775	-341.7	0.841	12.7
34	0.013682	-342.6	0.836	76.0	0.013682	-342.6	0.836	12.6
35	0.017588	-351.7	0.773	77.0	0.017588	-351.7	0.773	12.6
36	0.029306	-352.2	0.767	77.0	0.029306	-352.2	0.767	12.6

(continued on next page)

Table S11 (continued from previous page)

	DERNA				LinearCDSfold			
	λ_{DN}	MFE	CAI	Runtime (sec)	λ_{DN}	MFE	CAI	Runtime (sec)
37	0.037119	-352.6	0.760	77.0	0.037119	-352.6	0.760	12.6
38	0.060556	-352.6	0.760	77.0	0.060556	-352.6	0.760	12.7
39	0.072274	-352.9	0.751	77.0	0.072274	-352.9	0.751	12.6
40	0.080087	-353.0	0.748	77.0	0.080087	-353.0	0.748	12.7
41	0.138679	-353.2	0.740	77.0	0.138679	-353.2	0.740	12.7
42	0.000987	-257.3	0.988	76.0	0.000987	-257.3	0.988	12.6
43	0.000498	-235.7	0.997	76.0	0.000498	-235.7	0.997	12.6
44	0.001475	-269.0	0.980	76.0	0.001475	-269.0	0.980	12.6

Table S12: Distinct Pareto-optimal CDSs generated by DERNA and LinearCDSfold for protein sequence P0AC51. Both methods yielded the same set of 18 distinct Pareto-optimal CDSs.

	DERNA				LinearCDSfold			
Number	MFE	CAI	MFE (%)	CAI (%)	MFE	CAI	MFE (%)	CAI (%)
1	-225.5	1.000	0	100	-225.5	1.000	0	100
2	-353.4	0.726	100	0	-353.4	0.726	100	0
3	-353.2	0.740	100	5	-353.2	0.740	100	5
4	-352.9	0.751	100	9	-352.9	0.751	100	9
5	-352.2	0.767	99	15	-352.2	0.767	99	15
6	-350.7	0.781	98	20	-350.7	0.781	98	20
7	-352.6	0.760	99	12	-352.6	0.760	99	12
8	-353.0	0.748	100	8	-353.0	0.748	100	8
9	-340.5	0.846	90	44	-340.5	0.846	90	44
10	-351.7	0.773	99	17	-351.7	0.773	99	17
11	-318.5	0.908	73	66	-318.5	0.908	73	66
12	-342.6	0.836	92	40	-342.6	0.836	92	40
13	-285.8	0.963	47	86	-285.8	0.963	47	86
14	-336.1	0.860	86	49	-336.1	0.860	86	49
15	-341.7	0.841	91	42	-341.7	0.841	91	42
16	-257.3	0.988	25	96	-257.3	0.988	25	96
17	-235.7	0.997	8	99	-235.7	0.997	8	99
18	-269.0	0.980	34	93	-269.0	0.980	34	93

Table S13: Pareto-optimal CDSs generated by DERNA and LinearCDSfold for protein sequence Q8VIL3 (266 amino acids). Both methods explored 22 λ_{DN} values and yielded the same set of 13 distinct Pareto-optimal CDSs (see Supplementary Table S14). The total exploration times were 3,576 seconds for DERNA and 697 seconds for LinearCDSfold.

	DERNA				LinearCDSfold			
Index	λ_{DN}	MFE	CAI	Runtime (sec)	λ_{DN}	MFE	CAI	Runtime (sec)
1	0.000010	-313.6	1.000	161.0	0.000010	-313.6	1.000	31.5
2	0.999990	-498.3	0.771	163.0	0.999990	-498.3	0.771	31.7
3	0.500000	-498.3	0.771	163.0	0.500000	-498.3	0.771	31.7
4	0.250005	-498.3	0.771	163.0	0.250005	-498.3	0.771	31.7
5	0.125007	-498.3	0.771	163.0	0.125007	-498.3	0.771	31.8
6	0.062509	-498.3	0.771	163.0	0.062509	-498.3	0.771	31.7
7	0.031259	-497.2	0.789	163.0	0.031259	-497.2	0.789	31.8
8	0.015635	-497.2	0.789	163.0	0.015635	-497.2	0.789	31.8
9	0.046884	-497.2	0.789	163.0	0.046884	-497.2	0.789	31.7
10	0.007822	-482.7	0.848	162.0	0.007822	-482.7	0.848	31.6
11	0.054696	-497.2	0.789	163.0	0.054696	-497.2	0.789	31.8
12	0.003916	-443.7	0.922	162.0	0.003916	-443.7	0.922	31.6
13	0.011729	-484.2	0.843	163.0	0.011729	-484.2	0.843	31.8
14	0.058603	-498.3	0.771	163.0	0.058603	-498.3	0.771	31.7
15	0.001963	-405.8	0.957	162.0	0.001963	-405.8	0.957	31.6
16	0.005869	-471.2	0.873	162.0	0.005869	-471.2	0.873	31.6
17	0.009775	-483.9	0.844	162.0	0.009775	-483.9	0.844	31.8
18	0.013682	-487.5	0.831	163.0	0.013682	-487.5	0.831	31.7
19	0.056650	-498.3	0.771	163.0	0.056649	-498.3	0.771	31.7
20	0.000987	-346.1	0.994	162.0	0.000987	-346.1	0.994	31.7
21	0.000498	-336.0	0.997	162.0	0.000498	-336.0	0.997	31.7
22	0.001475	-355.7	0.989	162.0	0.001475	-355.7	0.989	31.6

Table S14: Distinct Pareto-optimal CDSs generated by DERNA and LinearCDSfold for protein sequence Q8VIL3. Both methods yielded the same set of 13 distinct Pareto-optimal CDSs.

	DERNA				LinearCDSfold			
Number	MFE	CAI	MFE (%)	CAI (%)	MFE	CAI	MFE (%)	CAI (%)
1	-313.6	1.000	0	100	-313.6	1.000	0	100
2	-498.3	0.771	100	0	-498.3	0.771	100	0
3	-497.2	0.789	99	8	-497.2	0.789	99	8
4	-482.7	0.848	92	34	-482.7	0.848	92	34
5	-443.7	0.922	70	66	-443.7	0.922	70	66
6	-484.2	0.843	92	32	-484.2	0.843	92	32
7	-405.8	0.957	50	81	-405.8	0.957	50	81
8	-471.2	0.873	85	45	-471.2	0.873	85	45
9	-483.9	0.844	92	32	-483.9	0.844	92	32
10	-487.5	0.831	94	26	-487.5	0.831	94	26
11	-346.1	0.994	18	97	-346.1	0.994	18	97
12	-336.0	0.997	12	99	-336.0	0.997	12	99
13	-355.7	0.989	23	95	-355.7	0.989	23	95

Table S15: Pareto-optimal CDSs generated by DERNA and LinearCDSfold for protein sequence O95229 (277 amino acids). Both methods explored 28 λ_{DN} values and yielded the same set of 14 distinct Pareto-optimal CDSs (see Supplementary Table S16). The total exploration times were 5,137 seconds for DERNA and 968 seconds for LinearCDSfold.

	DERNA				LinearCDSfold			
Index	λ_{DN}	MFE	CAI	Runtime (sec)	λ_{DN}	MFE	CAI	Runtime (sec)
1	0.000010	-367.9	1.000	182.0	0.000010	-367.9	1.000	34.5
2	0.999990	-557.0	0.806	184.0	0.999990	-557.0	0.806	34.6
3	0.500000	-557.0	0.806	184.0	0.500000	-557.0	0.806	34.6
4	0.250005	-557.0	0.806	184.0	0.250005	-557.0	0.806	34.6
5	0.125007	-557.0	0.806	184.0	0.125007	-557.0	0.806	34.5
6	0.062509	-557.0	0.806	184.0	0.062509	-557.0	0.806	34.5
7	0.031259	-556.0	0.818	184.0	0.031259	-556.0	0.818	34.6
8	0.015635	-554.3	0.827	184.0	0.015635	-554.3	0.827	34.5
9	0.046884	-556.6	0.812	184.0	0.046884	-556.6	0.812	34.6
10	0.007822	-536.3	0.880	183.0	0.007822	-536.3	0.880	34.5
11	0.023447	-556.0	0.818	184.0	0.023447	-556.0	0.818	34.4
12	0.039072	-556.6	0.812	184.0	0.039072	-556.6	0.812	34.5
13	0.054696	-557.0	0.806	184.0	0.054696	-557.0	0.806	34.5
14	0.003916	-517.5	0.918	183.0	0.003916	-517.5	0.918	34.7
15	0.011729	-552.9	0.832	184.0	0.011729	-552.9	0.832	34.5
16	0.019541	-556.0	0.818	184.0	0.019541	-556.0	0.818	34.5
17	0.035166	-556.0	0.818	184.0	0.035166	-556.0	0.818	34.6
18	0.050790	-557.0	0.806	184.0	0.050790	-557.0	0.806	34.4
19	0.001963	-462.9	0.968	182.0	0.001963	-462.9	0.968	34.7
20	0.005869	-523.6	0.908	183.0	0.005869	-523.6	0.908	34.7
21	0.009775	-547.3	0.850	183.0	0.009775	-547.3	0.850	34.6
22	0.013682	-554.3	0.827	184.0	0.013682	-554.3	0.827	34.6
23	0.017588	-556.0	0.818	183.0	0.017588	-556.0	0.818	34.6
24	0.037119	-556.6	0.812	184.0	0.037119	-556.6	0.812	34.6
25	0.048837	-557.0	0.806	184.0	0.048837	-557.0	0.806	34.7
26	0.000987	-418.6	0.990	182.0	0.000987	-418.6	0.990	34.5
27	0.000498	-393.4	0.997	182.0	0.000498	-393.4	0.997	34.5
28	0.001475	-443.8	0.980	182.0	0.001475	-443.8	0.980	34.5

Table S16: Distinct Pareto-optimal CDSs generated by DERNA and LinearCDSfold for protein sequence O95229. Both methods yielded the same set of 14 distinct Pareto-optimal CDSs.

	DERNA				LinearCDSfold			
Number	MFE	CAI	MFE (%)	CAI (%)	MFE	CAI	MFE (%)	CAI (%)
1	-367.9	1.000	0	100	-367.9	1.000	0	100
2	-557.0	0.806	100	0	-557.0	0.806	100	0
3	-556.0	0.818	99	6	-556.0	0.818	99	6
4	-554.3	0.827	99	11	-554.3	0.827	99	11
5	-556.6	0.812	100	3	-556.6	0.812	100	3
6	-536.3	0.880	89	38	-536.3	0.880	89	38
7	-517.5	0.918	79	58	-517.5	0.918	79	58
8	-552.9	0.832	98	13	-552.9	0.832	98	13
9	-462.9	0.968	50	84	-462.9	0.968	50	84
10	-523.6	0.908	82	53	-523.6	0.908	82	53
11	-547.3	0.850	95	23	-547.3	0.850	95	23
12	-418.6	0.990	27	95	-418.6	0.990	27	95
13	-393.4	0.997	13	99	-393.4	0.997	13	99
14	-443.8	0.980	40	90	-443.8	0.980	40	90

Table S17: Pareto-optimal CDSs generated by DERNA and LinearCDSfold for protein sequence Q2TBH8 (286 amino acids). Both methods explored 37 λ_{DN} values and yielded the same set of 19 distinct Pareto-optimal CDSs (see Supplementary Table S18). The total exploration times were 6,177 seconds for DERNA and 1,256 seconds for LinearCDSfold.

	DERNA				LinearCDSfold			
Index	λ_{DN}	MFE	CAI	Runtime (sec)	λ_{DN}	MFE	CAI	Runtime (sec)
1	0.000010	-364.5	1.000	166.0	0.000010	-364.5	1.000	34.0
2	0.999990	-546.5	0.780	167.0	0.999990	-546.5	0.780	33.8
3	0.500000	-546.5	0.780	168.0	0.500000	-546.5	0.780	34.2
4	0.250005	-546.5	0.780	168.0	0.250005	-546.5	0.780	33.9
5	0.125007	-546.5	0.780	167.0	0.125007	-546.5	0.780	33.9
6	0.062509	-545.7	0.804	167.0	0.062509	-545.7	0.804	33.8
7	0.031259	-543.7	0.833	167.0	0.031259	-543.7	0.833	33.9
8	0.093758	-545.7	0.804	167.0	0.093758	-545.7	0.804	33.8
9	0.015635	-538.0	0.863	167.0	0.015635	-538.0	0.863	33.8
10	0.046884	-544.3	0.826	167.0	0.046884	-544.3	0.826	34.0
11	0.109383	-546.5	0.780	167.0	0.109383	-546.5	0.780	33.9
12	0.007822	-533.3	0.881	167.0	0.007822	-533.3	0.881	34.1
13	0.023447	-543.7	0.833	167.0	0.023447	-543.7	0.833	33.9
14	0.039072	-544.1	0.829	167.0	0.039072	-544.1	0.829	33.9
15	0.054696	-545.7	0.804	167.0	0.054696	-545.7	0.804	34.0
16	0.101570	-546.5	0.780	167.0	0.101570	-546.5	0.780	33.9
17	0.003916	-507.3	0.923	166.0	0.003916	-507.3	0.923	33.8
18	0.011729	-534.9	0.876	167.0	0.011729	-534.9	0.876	33.8
19	0.019541	-543.7	0.833	167.0	0.019541	-543.7	0.833	34.0
20	0.035166	-543.8	0.832	168.0	0.035166	-543.8	0.832	33.8
21	0.042978	-544.3	0.826	168.0	0.042978	-544.3	0.826	34.0
22	0.050790	-544.5	0.823	167.0	0.050790	-544.5	0.823	34.1
23	0.097664	-545.7	0.804	167.0	0.097664	-545.7	0.804	34.0
24	0.001963	-464.5	0.965	166.0	0.001963	-464.5	0.965	33.8
25	0.005869	-530.1	0.888	167.0	0.005869	-530.1	0.888	33.9
26	0.009775	-533.3	0.881	167.0	0.009775	-533.3	0.881	34.1
27	0.013682	-536.3	0.870	167.0	0.013682	-536.3	0.870	33.9
28	0.017588	-542.5	0.839	167.0	0.017588	-542.5	0.839	34.2
29	0.033213	-543.8	0.832	167.0	0.033212	-543.8	0.832	34.1
30	0.037119	-544.1	0.829	167.0	0.037119	-544.1	0.829	34.2
31	0.041025	-544.1	0.829	167.0	0.041025	-544.1	0.829	34.0
32	0.048837	-544.5	0.823	167.0	0.048837	-544.5	0.823	33.9
33	0.052743	-544.5	0.823	167.0	0.052743	-544.5	0.823	34.1
34	0.099617	-546.5	0.780	167.0	0.099617	-546.5	0.780	34.2
35	0.000987	-415.8	0.993	166.0	0.000987	-415.8	0.993	33.9
36	0.000498	-396.0	0.998	166.0	0.000498	-396.0	0.998	33.9
37	0.001475	-427.4	0.988	166.0	0.001475	-427.4	0.988	33.8

Table S18: Distinct Pareto-optimal CDSs generated by DERNA and LinearCDSfold for protein sequence Q2TBH8. Both methods yielded the same set of 19 distinct Pareto-optimal CDSs.

	DERNA				LinearCDSfold			
Number	MFE	CAI	MFE (%)	CAI (%)	MFE	CAI	MFE (%)	CAI (%)
1	-364.5	1.000	0	100	-364.5	1.000	0	100
2	-546.5	0.780	100	0	-546.5	0.780	100	0
3	-545.7	0.804	100	11	-545.7	0.804	100	11
4	-543.7	0.833	98	24	-543.7	0.833	98	24
5	-538.0	0.863	95	38	-538.0	0.863	95	38
6	-544.3	0.826	99	21	-544.3	0.826	99	21
7	-533.3	0.881	93	46	-533.3	0.881	93	46
8	-544.1	0.829	99	22	-544.1	0.829	99	22
9	-507.3	0.923	78	65	-507.3	0.923	78	65
10	-534.9	0.876	94	44	-534.9	0.876	94	44
11	-543.8	0.832	99	24	-543.8	0.832	99	24
12	-544.5	0.823	99	20	-544.5	0.823	99	20
13	-464.5	0.965	55	84	-464.5	0.965	55	84
14	-530.1	0.888	91	49	-530.1	0.888	91	49
15	-536.3	0.870	94	41	-536.3	0.870	94	41
16	-542.5	0.839	98	27	-542.5	0.839	98	27
17	-415.8	0.993	28	97	-415.8	0.993	28	97
18	-396.0	0.998	17	99	-396.0	0.998	17	99
19	-427.4	0.988	35	94	-427.4	0.988	35	94

References

[1] X. Y. Gu et al. DERNA enables Pareto optimal RNA design. *Journal of Computational Biology*, 31:179–196, 2024.

[2] Y. R. Ju et al. A more efficient dynamic programming algorithm for designing a coding sequence by jointly optimizing its structural stability and codon usage. *IEEE Transactions on Computational Biology and Bioinformatics*, 2025. doi: 10.1109/TCBBIO.2025.3596771.
